# Targeting the Ophthalmic Diseases Using Extracellular Vesicles ‘Exosomes’: Current Insights on Their Clinical Approval and Present Trials

**DOI:** 10.14336/AD.2024.0535

**Published:** 2025-05-25

**Authors:** Hanxiang Yu, Jinsong Wu, Gaoxiang Pan

**Affiliations:** ^1^Queen Marry College, Nanchang University, Nanchang, Jiangxi 330006, China.; ^2^Department of Pediatric Ophthalmology, The Second Affiliated Hospital of Nanchang University, Nanchang, Jiangxi 330006, China.; ^3^Queen Marry College, Nanchang University, Nanchang, Jiangxi 330006, China.

**Keywords:** Ophthalmic diseases, Exosomes, Clinical trials, Drug targeting, Drug Delivery

## Abstract

Ophthalmic diseases encompass a diverse range of conditions, each necessitating tailored treatment strategies. In the realm of ophthalmic research and therapeutic interventions, various subtypes of exosomes are being explored for their regenerative, neuroprotective, and anti-inflammatory properties. Exosomes have garnered increasing attention as promising therapeutic vehicles due to their natural role in cell-to-cell communication and targeted delivery capabilities. Derived from cells, these small vesicles facilitate the transportation of numerous molecules between cells, offering advantages such as low immunogenicity, stability, and precise cell targeting. These inherent qualities make exosomes an enticing avenue for advancing treatment options for ophthalmic diseases. While ongoing research and clinical applications continue to evolve, several exosome subtypes have demonstrated potential for addressing various ophthalmic conditions, including glaucoma, age-related macular degeneration, retinal degenerative disorders, and ocular inflammatory conditions.

## Introduction

**Figure 1.**
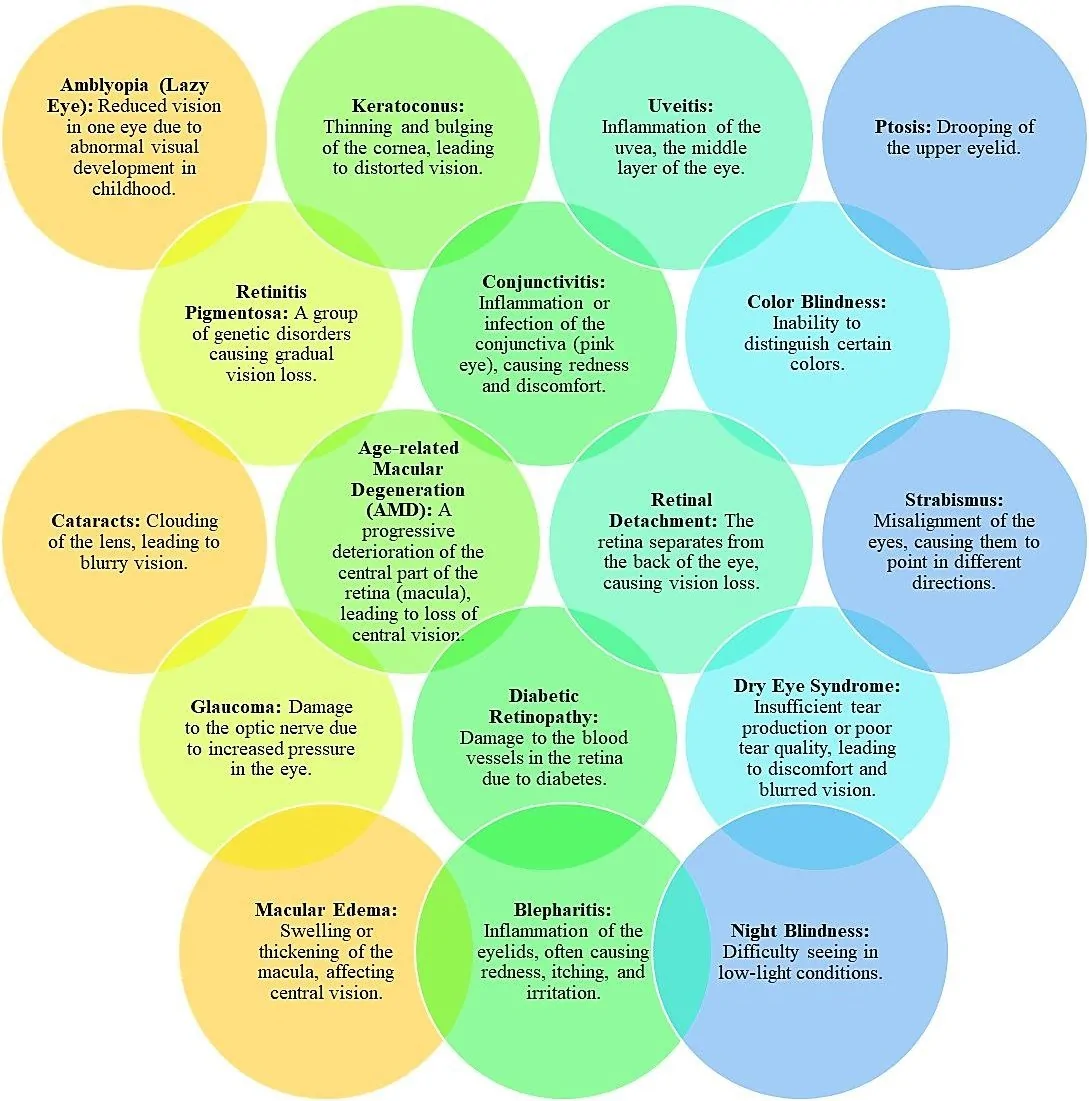
Common eye conditions and associated pathophysiology.

Ophthalmic diseases cover a wide range of conditions affecting the eyes and their structures, potentially leading to vision impairment or blindness. These ailments vary in severity and require different treatments, such as medications, corrective lenses, surgery, or a combination of approaches [1]. The prevalence of all other ocular manifestations, except for retinal vaso-occlusive disease, was consistently below 5% [2]. Common eye conditions encompass a range of ailments: cataracts, which cause blurry or reduced clarity vision by clouding the eye lens; glaucoma, marked by heightened eye pressure leading to optic nerve damage and potential irreversible vision loss; macular degeneration, impacting central vision through macular impairment; diabetic retinopathy, a diabetic complication damaging retinal blood vessels, often resulting in vision impairment or blindness; refractive errors like myopia, hyperopia, astigmatism, and presbyopia, necessitating corrective lenses or surgery; conjunctivitis, commonly known as "pink eye," inducing swelling in the eye's clear surface tissue; retinal detachment, a critical medical emergency where the retina detaches from underlying layers, requiring immediate attention to prevent permanent vision loss; strabismus, characterized by misaligned eyes affecting depth perception and potentially causing amblyopia if untreated in childhood; and dry eye syndrome, stemming from insufficient tear production or poor-quality tears, leading to discomfort and vision disturbances [2-4]. Uveitis, characterized by inflammation of the uvea, may present symptoms such as pain, redness, and vision alterations. To safeguard vision and ocular health, a systematic approach is crucial for early detection and efficient management of these ophthalmic conditions [5, 6]. The common eye diseases and their succinct pathophysiology are illustrated in Figure 1.

Ophthalmic diseases include a wide array of conditions, each requiring individualized treatment approaches. Medications, such as eye drops, ointments, or oral drugs, are common approach for various eye ailments. Antibiotics combat infections, anti-inflammatory medications alleviate conditions like uveitis, and eye pressure-lowering drugs help control glaucoma. Surgical interventions are vital in treating several ophthalmic disorders. Cataract removal surgery involves swapping the cloudy lens with an artificial one, while procedures like retinal detachment repair or glaucoma surgery address structural issues [7, 8]. Laser therapy, frequently employed in ophthalmology, is instrumental in managing diabetic retinopathy by sealing abnormal blood vessels and in certain cases of glaucoma treatment. Injections administered directly into the eye, utilized for conditions such as macular degeneration or diabetic retinopathy, facilitate precise medication delivery. Additionally, vision correction options such as glasses, contacts, or refractive surgeries like LASIK effectively address refractive errors [9]. Systemically administered drugs have limited effectiveness in the eye due to poor blood flow in the corneal cells and tissues. In some cases, injecting the drug into the eye cavity is recommended to target the posterior region, but this method is painful and often leads to patient noncompliance. Rapid drainage of topically applied drugs reduces their pharmacological action, necessitating increased dosing frequency. Additionally, the portion of the drug that enters systemic circulation through various routes can cause systemic toxic effects [8]. Thus, there is need new treatments for eye diseases.

Lifestyle adjustments, such as diabetes management to prevent diabetic retinopathy, complement treatment regimens. Specialized therapies for eye alignment issues, coupled with cutting-edge technologies like retinal implants, broaden the spectrum of treatment options. Consistent monitoring and follow-up appointments ensure treatment effectiveness and timely adaptations for optimal outcomes, underscoring the significance of tailored care in managing ophthalmic conditions [10]. Seeking guidance from an ophthalmologist is pivotal for precise diagnosis and the formulation of individualized treatment plans tailored to each patient's unique requirements [11].

**Figure 2.**
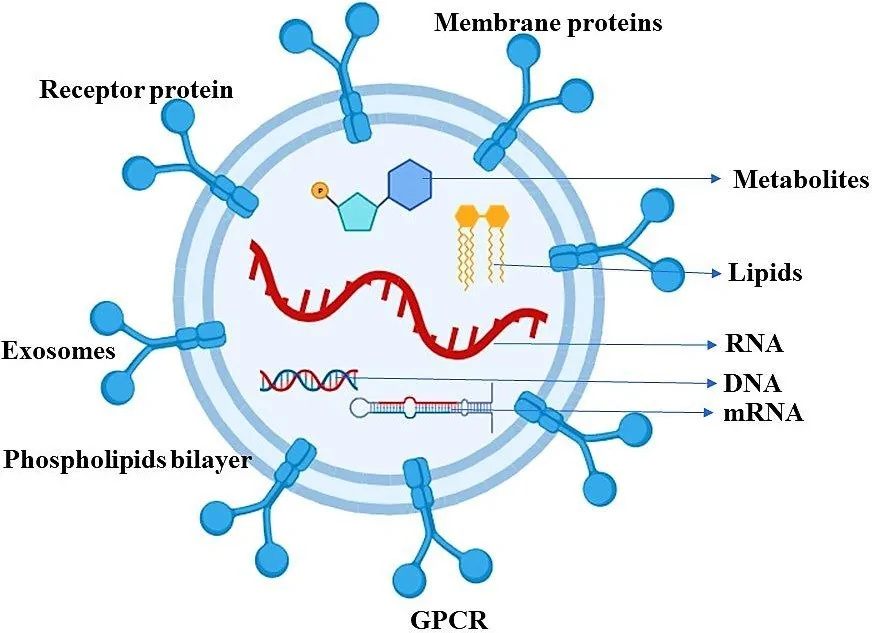
Illustration to portray the composition of the exosomes which includes a plethora of proteins, nucleic acids, lipid components, and metabolites.

Exosomes, minuscule vesicles secreted by cells, possess distinctive biological properties with therapeutic potential. These nanoscale structures, abundant in proteins, lipids, RNA, and DNA, serve as mediators in intercellular communication. Illustrated in Figure 2 are exosomes and their principal components. Their biocompatibility and capacity for targeted delivery to specific cells or tissues afford them an advantage over conventional drugs [12].

Initial proteomic studies revealed that exosomes contain a specific subset of proteins from endosomes, the plasma membrane, and the cytosol, but very few from other intracellular organelles such as the nucleus, mitochondria, and Golgi apparatus. Exosomes are a subtype of secreted vesicles formed inside eukaryotic cells within multivesicular compartments. These compartments fuse with the plasma membrane to release exosomes. Interestingly, different families of molecules facilitate both the intracellular formation and secretion of exosomes, suggesting that there are various subtypes of exosomes. Exosomes are small vesicles with a lipid bilayer containing various proteins, RNAs, and bioactive lipids. They function as intercellular messengers, enabling communication between cells of the same type and different types. Released by healthy cells both constitutively and upon activation, exosomes play a crucial role in immune system function. While essential for maintaining healthy physiological conditions, exosomes can also potentiate cellular stress and damage under pathological circumstances [12, 13].

Moreover, exosomes hold significant promise for application in regenerative medicine due to their rich content of signaling molecules capable of stimulating tissue repair and modulating immune responses. Their inherent stability safeguards their cargo from degradation, minimizing off-target effects and adverse reactions [13]. While challenges persist in exosome research, such as standardizing isolation methods and enhancing production, ongoing research endeavors seek to surmount these obstacles and deepen our understanding of exosome mechanisms of action. With potential applications in personalized medicine, regenerative therapies, cancer treatment, and precise drug delivery, exosomes offer a promising outlook [14].

Exosomes, derived from various cellular sources including retinal or stem cells, exhibit remarkable efficacy in treating ocular diseases. They demonstrate effectiveness in managing retinal conditions like age-related macular degeneration (AMD) and retinal degenerative disorders, slowing degeneration, and preserving retinal function. In corneal healing, preclinical studies suggest promise for exosomes derived from mesenchymal stem cells (MSCs), as they reduce inflammation, stimulate cell proliferation, and facilitate tissue regeneration [15, 16].

Furthermore, exosomes exhibit neuroprotective potential in glaucoma, a condition necessitating neuroprotection. They may create a protective milieu for retinal ganglion cells, mitigating harm and degeneration and potentially altering disease progression. In ocular therapeutics, exosomes present an appealing option for drug delivery owing to their natural carrier properties [17]. Researchers are exploring their capacity to transport therapeutic agents or genetic material to target specific ocular cells, potentially enhancing treatment efficacy and reducing adverse effects [18].

However, despite these promising avenues, hurdles persist in harnessing exosomes for clinical use in ophthalmology. Critical challenges include standardizing isolation techniques, elucidating their precise mechanisms of action in ocular tissues, and ensuring scalability for clinical applications. As research advances, the therapeutic potential of exosomes in managing ocular diseases represents an exciting frontier, offering innovative and targeted interventions for various eye conditions in the future.

Regarding the importance and efficiency of exosomes in treating eye diseases, we could not find a comprehensive study investigating their effects on ophthalmic conditions. Numerous studies have determined the potential of exosomes in ophthalmology owing to their capacity to deliver therapeutic cargo to specific target cells in the eye. These studies have explored exosome-based therapies for circumstances such as age- related macular degeneration (AMD), diabetic retinopathy, retinal ischemia, and corneal injuries. Therefore, this study aims to review and target the potential use of exosomes in ophthalmic diseases.

## Exosomes and their subtypes

Exosomes, minute vesicles secreted by cells, exhibit diverse classifications based on their origin, content, and functions:

### Endosomal-Derived Exosomes

Formed within multivesicular bodies (MVBs) through the endosomal pathway, these exosomes are released upon MVB fusion with the cell membrane. They ferry a range of molecules, including proteins, lipids, and genetic material, serving as crucial mediators of intercellular communication [19, 20].

### Dendritic Cell-Derived Exosomes

Secreted by dendritic cells, these exosomes contain proteins and molecules that modulate the immune system. They participate in antigen presentation, immune responses, and hold potential for immunotherapeutic applications [21].

### Stem Cell-Derived Exosomes

Encompassing a diverse array of pharmacologically active molecules, growth factors, and RNA, exosomes derived from stem cells exhibit regenerative properties and are investigated for therapeutic utility across various diseases [22].

### Tumor-Derived Exosomes

Emitted by cancer cells, these exosomes harbor specific proteins, nucleic acids, and other molecules that facilitate tumor growth, metastasis, and immune modulation, influencing cancer progression [23].

### Plasma Derived Exosomes

Present in blood plasma, these exosomes carry cargo from various cell types within the body. They are considered potential biomarkers for diverse diseases owing to their accessibility in bodily fluids [24, 25].

### Neuronal Exosomes

Released by neurons, these exosomes contain proteins, lipids, and genetic material involved in synaptic function and neuronal communication. They are implicated in neurological disorders such as Alzheimer's and Parkinson's diseases [26].

### Cardiac Exosomes

Released by cardiac cells, cardiac exosomes transport molecules essential for cardiac function, repair, and stress resilience. They hold promise for cardiac repair and serve as diagnostic markers [27].

### Adipose-Derived Exosomes

Originating from fat tissue, these exosomes contain factors associated with metabolism, inflammation, and tissue repair. They are being explored for their regenerative potential in wound healing and tissue regeneration [28].

### Synaptic Exosomes

Released at synapses, synaptic exosomes regulate synaptic activity and plasticity, influencing communication between neurons and impacting learning and memory processes [29, 30].

### Microglial Exosomes

Originating from microglia, these exosomes contain molecules related to immune responses and neuroinflammation, with potential applications in neurodegenerative diseases and brain injury [31].

### Bone-Derived Exosomes

Produced by bone cells such as osteoblasts and osteoclasts, these exosomes carry factors involved in bone remodeling, mineralization, and intercellular communication, contributing to bone health and potentially useful in conditions like osteoporosis [32].

### Immune Cell-Derived Exosomes

Secreted by immune cells like B cells, T cells, and macrophages, these exosomes contain proteins and molecules crucial for immune regulation, antigen presentation, and intercellular signaling, impacting immune responses [33].

### Hepatic Exosomes

Secreted by liver cells, these exosomes carry molecules associated with liver function, metabolism, and intrahepatic communication, potentially playing roles in liver diseases and metabolic disorders [34].

### Gut-Derived Exosomes

Originating from cells in the gastrointestinal tract, these exosomes contain molecules related to gut health, digestion, and communication within the gut microbiota, influencing gut homeostasis and gastrointestinal diseases [35, 36].

### Skin-Derived Exosomes

Produced by skin cells like keratinocytes and fibroblasts, these exosomes contain factors promoting skin regeneration, wound healing, and intercellular communication in the skin [37].

### Pancreatic Exosomes

Originating from pancreatic cells, these exosomes contain molecules affecting insulin secretion, glucose regulation, and intra-pancreatic communication, potentially impacting conditions like diabetes and pancreatic disorders [38].

### Reproductive Cell-Derived Exosomes

Released by reproductive cells like sperm and ovarian cells, these exosomes carry genetic material and factors involved in reproduction, potentially influencing fertility, embryo development, and reproductive health [39].

### Exosomes in Extracellular Matrix Regulation

Associated with the extracellular matrix, these exosomes contain cargo affecting extracellular matrix remodeling, cell adhesion, and tissue architecture, contributing to tissue repair and regeneration processes [40].

### Viral Exosomes

Produced by viruses, these exosomes contain viral components facilitating viral spread, immune evasion, and modulation of host cell responses, influencing viral pathogenesis [41].

### Exercise-Induced Exosomes

Stimulated by physical exercise, these exosomes carry factors mediating some of the beneficial effects of exercise on other tissues and organs [42].

### Hematopoietic Stem Cell-Derived Exosomes

Originating from hematopoietic stem cells, these exosomes carry factors involved in blood cell formation, immune modulation, and tissue repair, with implications in hematological disorders and immune-related conditions [43, 44].

### Adeno-Associated Virus (AAV) Exosomes

AAVs used as gene therapy vectors can generate exosomes containing therapeutic genetic material, holding potential for delivering genetic payloads to target cells and tissues for therapeutic purposes [45, 46].

**Figure 3.**
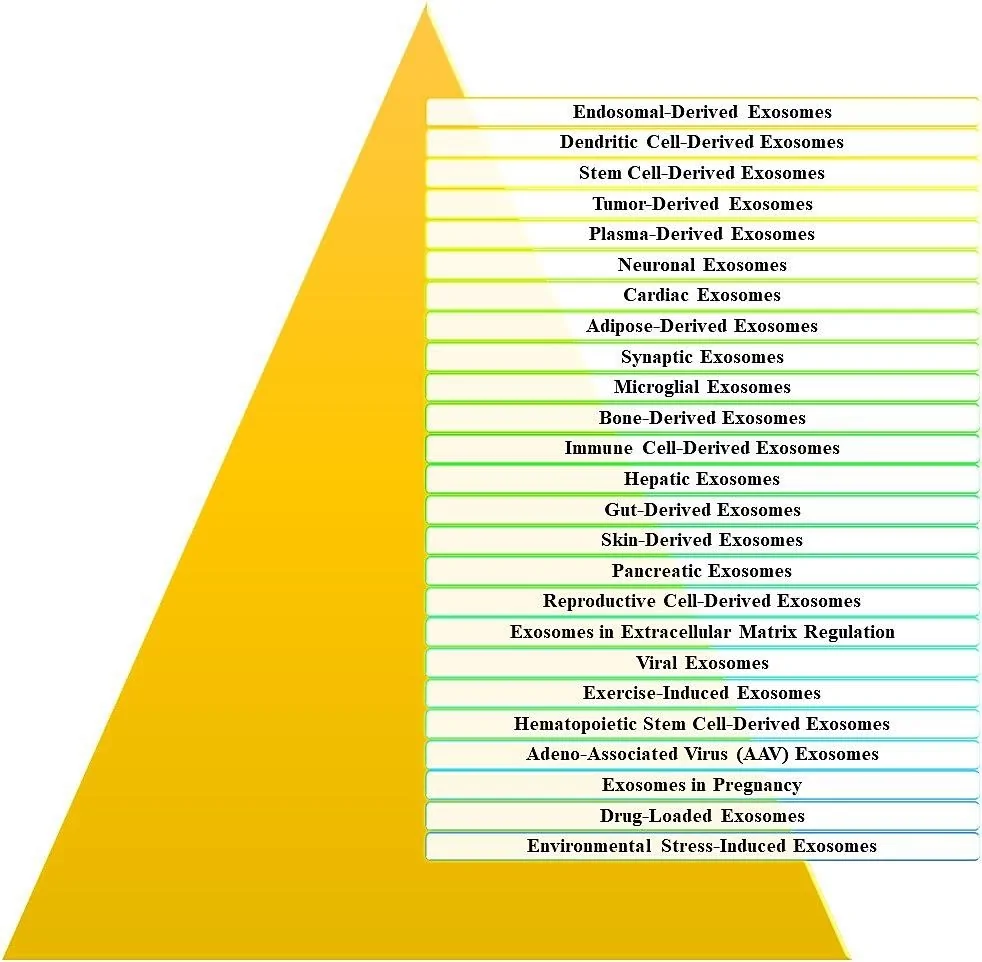
Illustration of some exosomes that are commonly used in the treatment of ophthalmic disorders.

In ophthalmic research and therapy for potential pharmacological indications, several subtypes of exosomes are under exploration for their regenerative, neuroprotective, and anti-inflammatory characteristics. While ongoing research and emerging clinical applications continue, various exosome subtypes show promise for addressing ophthalmic diseases such as retinal degenerative diseases, age-related macular degeneration, glaucoma, and ocular inflammatory conditions. Stem cell-derived exosomes, including those from mesenchymal stem cells (MSCs), demonstrate regenerative potential. Neuronal cell-derived exosomes are investigated for their role in neuroprotection [47, 48]. Dendritic cell-derived exosomes, known for immune regulatory properties, exhibit potential in modulating immune responses in ocular inflammatory conditions [49]. Plasma-derived exosomes found in blood plasma are explored as possible biomarkers for ocular diseases, aiding in diagnostics and disease monitoring. Finally, exosomes from various cell types, including those associated with the extracellular matrix, are under exploration for their potential roles in supporting tissue repair and modulating inflammatory responses in ocular tissues [50]. This section provides a comprehensive review of exosomes and their subtypes, as well as their efficiencies for clinical use (Fig. 3).

## 3. Biological mechanism of exosomes: Understanding their functioning

### Biogenesis and Vesicle Sorting

Exosomes undergo a tightly regulated process within the endosomal system. Intraluminal vesicles (ILVs) accumulate as the endosomal membrane invaginates during the maturation of early endosomes into late endosomes or multivesicular bodies (MVBs). These ILVs encapsulate a diverse array of proteins, lipids, nucleic acids, and metabolites. Specific mechanisms, including the ESCRT (endosomal sorting complexes required for transport) machinery, tetraspanins, ceramides, and various RNA-binding proteins, facilitate cargo selection and packaging into ILVs, contributing to the unique composition of exosomes [12].

### Release and Uptake Mechanisms

Once formed, MVBs can either fuse with lysosomes for degradation or fuse with the cell membrane, releasing ILVs as exosomes. These exosomes interact with target cells via various mechanisms. They can bind to cell surface receptors, leading to receptor-mediated endocytosis or phagocytosis. Alternatively, exosomes may directly fuse with the recipient cell's plasma membrane, releasing their cargo into the cytoplasm or endosomal compartments. The uptake mechanisms vary depending on cell type and exosome cargo [13].

### Functional Impact

Exosome cargo confers diverse functionalities upon recipient cells. They participate in cell-to-cell communication by transferring bioactive molecules that modulate gene expression, cellular signaling pathways, and functional phenotypes of target cells. For example, exosomal microRNAs regulate gene expression by suppressing target mRNA translation, influencing various cellular processes. Similarly, exosomal proteins can trigger signaling cascades or alter recipient cell phenotypes, impacting their behavior or function [13].

### Exosome Stability and Physiological Barriers

Exosomes exhibit stability in biological fluids due to their lipid bilayer, protecting them from degradation by enzymes or environmental factors. This stability enables exosomes to transport functional molecules, facilitating communication between cells and tissues over long distances. Their ability to traverse physiological barriers and reach distant sites allows for systemic responses and potential effects on tissues remote from their origin [51].

**Figure 4.**
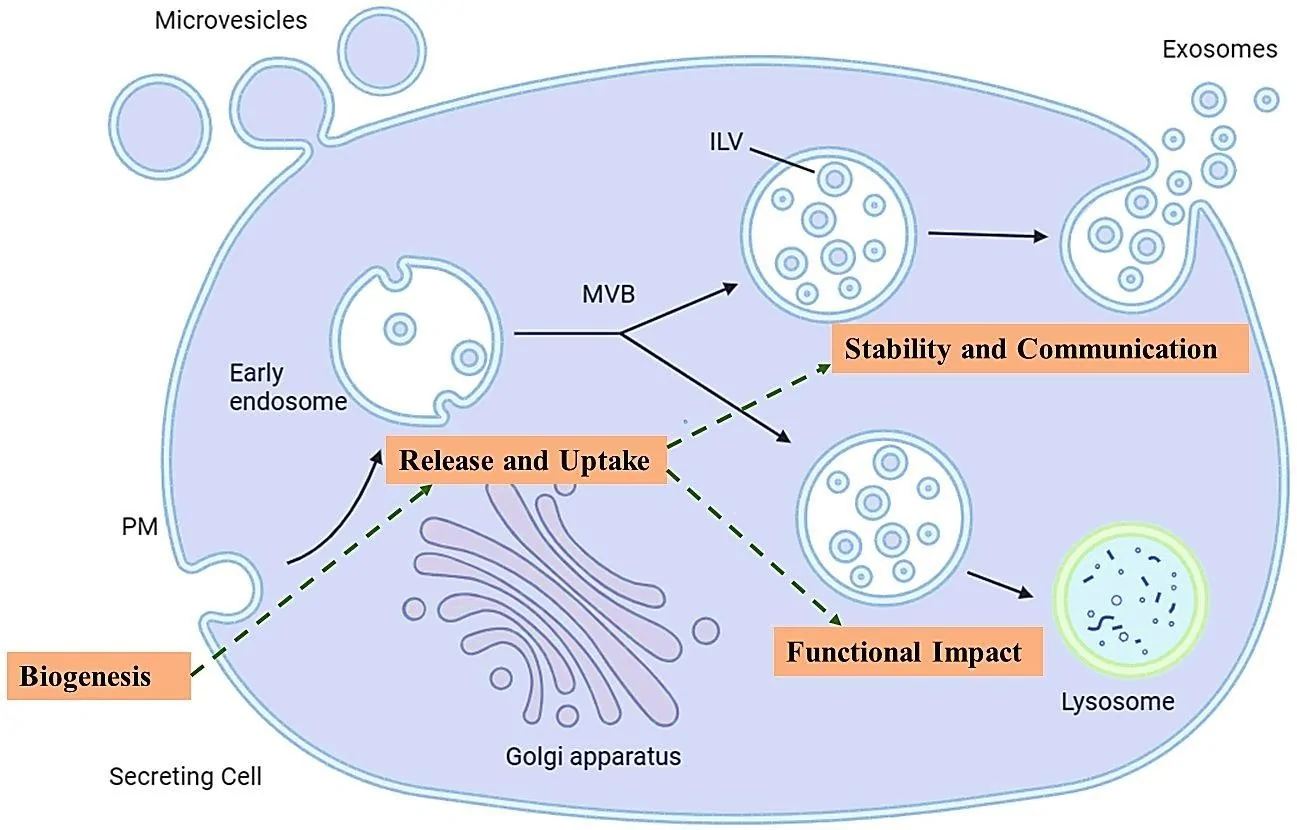
**The biological mechanism and functioning of exosomes can be illustrated through a series of intricate steps**. It all starts with biogenesis, initiating a cascade of events that lead to endosomal maturation, the formation of intraluminal vesicles (ILVs), and cargo sorting within multivesicular bodies (MVBs). Subsequently, the process branches into two main pathways: Release and Uptake. In one pathway, MVBs fuse with the cell membrane, facilitating the release of exosomes into the extracellular space. Simultaneously, another pathway diverges, wherein exosomes interact with recipient cells, initiating a cascade of events with Functional Impact. This impact includes alterations in gene expression, modulation, and cellular signaling. These alterations contribute to the Stability and Communication of exosomes, ensuring their role in long-distance communication and maintaining stability within the extracellular environment.

### Implications and Future Directions

Understanding the intricate mechanisms of exosome biogenesis, cargo sorting, release, and uptake provides insights into their roles in normal physiology and disease. Exosomes play a crucial role in intercellular communication, influencing various cellular pathways, and offering potential applications in diagnostics, therapeutics, and regenerative medicine. Ongoing research into exosome biology continues to uncover their remarkable potential for clinical and research applications across a wide range of biological and medical fields [52]. In this section, several mechanisms for the exosomes were mentioned that can help in the treatment of diseases and clinical uses. The brief mechanism and functioning of exosome are depicted in Figure 4.

## Categorized use of exosomes in different ophthalmic disease

Exosomes, small vesicles secreted by cells, hold promise in various ophthalmic diseases due to their ability to transfer molecular cargo, including proteins, lipids, and nucleic acids, between cells. Below is a categorized overview of exosome use in different ophthalmic diseases:

Age-related Macular Degeneration (AMD): Currently, anti-vascular endothelial growth factor therapy is the latest developed therapy for visual impairment in AMD. However, this therapy exhibits ineffectiveness in advanced stages of AMD, with some cases reporting severe vision loss post-treatment. Therapeutic applications of exosomes in AMD are detailed in Table 1 [53]. Exosomes possess advantages over other drug carriers, such as their nanoscale size, lower immunogenic behavior, higher cellular uptake due to longer circulation time, and low biodegradation. Exosomes have been investigated for their potential to deliver therapeutic molecules (such as miRNAs and anti-inflammatory agents) to target cells in the retina to mitigate AMD progression. Drug-loaded exosomes have shown improved efficacy and bioavailability compared to free drugs [55, 56].

**Table 1:** Therapeutic applications of exosomes in AMD.

Exosomes source cells	Exosome content	Target	Mechanism of action	Reference
**Mesenchymal** **Stem cell (MSC)**	---	Nrf2 signaling pathway	Improve the ratio of Bcl-2/Bax by inhibiting cell	Tang et al. [57]
			apoptosis and regulation of Nrf2 signaling pathway	
**Human** **umbilical cord blood MSCs (hUCMSCs)**	---	VEGF-A	Downregulation of VEGF-A resulting in better visual function via improving histological structures of CNV	He et al. [58]
**Human** **umbilical cord blood MSCs (hUCMSCs)**	miR-27b-3p	Reduction of retinal fibrosis	Suppress the epithelial-mesenchymal transition (EMT) in RPE cells	Li et al. [59]
**Human** **umbilical cord blood MSCs (hUCMSCs)**	miR-126	HMGB1 signaling pathway	Suppression of high glucose-mediated overexpressed HMGB1 activity in rat models	Zhang et al. [60]
**Retinal** **astroglial cells (RACs)**	Different antiangiogenic factor such as endostatin	Angiogenesis inhibitor	Suppress the leakage of retinal vessels and inhibition of CNV	Hajrasouliha et al. [61]

Tang et al. explored MSC-exosomes for the underlying mechanism in dry AMD and demonstrated that these exosomes protect retinal pigment epithelial (RPE) cells from oxidative stress by regulating the Nrf2 signaling pathway and inhibiting cellular apoptosis via improving the ratio of Bcl-2/Bax. Immunofluorescent studies showed that pre-administration of MSC exosomes protected the retina from NaIO3-induced damage [57]. He et al. isolated human umbilical cord blood MSCs (hUCMSCs)-derived exosomes rich in CD63 and CD90 markers. In vitro studies demonstrated that these exosomes significantly downregulated mRNA levels and VEGF-A protein expression in blue light-stimulated RPE cells. In vivo models showed that these MSC-derived exosomes reduced damage by downregulating VEGF-A, leading to improved visual functionality via enhanced histological structures of choroidal neovascularization (CNV) [58]. These results were further confirmed by Li and his group [59]. In advanced studies, Li and his group delivered miR-27b-3p via hUCMSCs-derived exosomes and observed suppression of the epithelial-mesenchymal transition (EMT) in RPE cells. EMT was induced by inhibiting HOXC6 expression and transforming growth factor-beta2 (TGF-β2) [59]. Zhang et al. transferred microRNA-126 (miR-126) using hUCMSCs-derived exosomes as carriers in hyperglycemia-induced retinal inflammatory rat models and successfully suppressed inflammation by inhibiting the HMGB1 signaling pathway. In vitro evaluations showed that miR-126-containing hUCMSCs-derived exosomes suppressed NLRP3 inflammasome activity in high glucose-affected human retinal endothelial cells (HRECs) [60]. Hajrasouliha et al. reported that retinal astrocytes-derived exosomes, rich in multiple antiangiogenic factors such as endostatin, inhibited angiogenesis and laser-induced CNV [61].

### Corneal Diseases

Exosomes have demonstrated potential in corneal wound healing and regeneration by delivering growth factors, cytokines, or miRNAs to promote corneal cell proliferation and tissue repair. Adipose stem cell-derived exosomes have acted as potential vehicles to deliver miRNA-19A, significantly suppressing corneal keratocyte differentiation by lowering HIPK2, phosphorylated Smad-3, p53, collagen III, and fibronectin levels [62]. In another study, adipose-derived mesenchymal stem cell-based exosomes, rich in miRNA-24-3p, reversed corneal injury in a rabbit model and inhibited corneal fibrosis and keratitis [63].

Zhou et al. revealed that bone marrow-derived MSCs-based exosomes activate the p44/42 MAPK pathway, downregulating fibrosis (α-SMA) and vascularization (CD31) in the cornea and reversing alkali-burn-induced injury in mouse models [64]. Similar results with bone marrow-derived MSCs-based exosomes were confirmed by Saccu et al., showing that administration of these exosomes creates an anti-inflammatory and pro-survival environment to prevent angiogenesis in corneal tissue [65]. Adipose-derived MSC-based exosomes are rich sources of various miRNAs. Shen et al. explored adipose-derived MSC-based exosomes and found that they downregulate matrix metalloproteins (MMP) in corneal stromal cells (CSCs) and enhance cell growth, with a small upregulation observed in extracellular matrix protein expression [66].

Samaeekia et al. explored human corneal MSC-derived exosomes for wound healing in corneal cells. CD marker-rich exosomes accelerated corneal wound healing in human corneal epithelial cells (HCECs) by over 50% compared to the control group (P < 0.005). In vivo studies also demonstrated similar results, highlighting the therapeutic potential of these exosomes in corneal epithelial wound healing [67]. Liu et al. delivered miRNA-21 using human umbilical cord MSC-derived exosomes, accelerating corneal wound healing by activating the PI3K/Akt pathways and decreasing PTEN levels. These exosomes served as potential carriers for miRNA-21, along with exosome markers [68].

The presence of miRNA-17-92 in bone marrow MSC-derived exosomes serves as a major therapeutic agent in suppressing PTEN and promoting RGCs survival and growth [69]. Table 2 lists the therapeutic potential of various exosomes in corneal diseases.

**Table 2:** Therapeutic potential of various exosomes in corneal diseases.

Exosomes source cells	Exosome content	Target	Mechanism of action	Reference
**Bone marrow- derived MSC**	----	p44/42 MAPK pathway	Activation of p44/42 MAPK pathway and	Zhou *et al.* 64
			Downregulated the α-SMA and CD31	
**Bone marrow- derived MSC**	----	Anti- inflammation	Modulate corneal hemostasis via providing anti- inflammatory effect and enhance cell survival	Saccu *et al.* 65
**Adipose- derived MSC**	----	Matrix metalloproteins (MMP)	Downregulate the MMP and upregulated extracellular matrix protein and fibronectin	Shen *et al.* 66
**Human corneal MSC**	----	----	Expedite *in vitro* and *in vivo* the corneal epithelial wound healing process	Samaeekia *et al.* 67
**Human** **umbilical cord MSC**	miRNA-21	PI3K/AKT and PTEN	Activation of PI3K/AKT pathways and decreased PTEN level	Liu *et al.* 68

Glaucoma: Glaucoma involves the degeneration of ganglion cells of the retina and axons, resulting in optic disc deformation and eventual vision loss [70, 71]. Current therapies aimed at lowering intraocular pressure, such as surgery, laser therapy, or hypotensive drops, are inadequate for reversing retinal ganglion cell (RGC) degeneration [72]. Studies suggest that exosomes play a role in transferring neuroprotective factors to RGCs, potentially aiding in preserving optic nerve function in glaucoma [73]. The delivery of therapeutics using exosomes as vesicles or carriers has shown significant results compared to naked therapeutics (Table 3 contains therapeutic applications of exosomes in Glaucoma).

**Table 3:** Therapeutic applications of exosomes in Glaucoma.

Exosomes source cells	Exosome content	Target	Mechanism of action	Reference
**HEK293T cells**	S58 aptamer	----	Antifibrotic, Inhibit the migration and cell proliferation in TGF-β2- induced HConFs	Lin *et al.* [74]
**Bone marrow- derived MSC (BMSC)**	MiRNA cargo such as MIR- 106A-5P, MIR- 486-5P, MIR-144- 5P, etc.	miRNA	miRNA effector molecule Argonaute-2 modulated therapeutic effect in ONC model	Mead and Tomarev [75, 76]
**Bone marrow- derived MSC (BMSC)**	Primed with TNFα or retinal conditioned medium	TNFα signaling	Protect hRGCs and retinal cells in rats via modulating TNFα signaling and improving PEDF and VEGF-AA levels	Mead *et al.* [77]
**Human embryonic- MSCs (ES- MSCs)**	----	Cis-p-tau and tauopathy	Reduce the level of cis-p-tau and block the tauopathy process in ONC model	Seyedrazizadeh *et al.* [78]
**Amniotic membrane** **mesenchymal (AMMSCs) andepithelial Stem cells (AMESCs)**	Higher levels of FGF, EGF, TGF- β, VEGF, BDNF and PDGF	NeuN	Improved level of NeuN biomarker in RGCs	Seong *et al.* [79]

In one study, exosome-mediated delivery of the S58 aptamer substantially demonstrated an antifibrotic effect and prolonged filtering bleb retention by inhibiting migration and cell proliferation in TGF-β2-induced human conjunctival fibroblasts (HConFs) compared to naked S58 [74].

Mead and Tomarev isolated exosomes from bone marrow-derived MSCs (BMSCs) and evaluated their therapeutic potential against a rat optic nerve crush (ONC) model. In retinal culture studies, BMSC-derived exosomes showed substantial neuroprotective and neuritogenic potential. In 21-day studies, significant survival of RGCs along with axon regeneration was observed [75]. Intravitreal injection of BMSC-derived exosomes in DBA/2J mice (3 months old) preserved RGC function up to 6 months of age and reduced optic nerve axon degeneration, with no effects observed in 9 and 12-month-old mice [76]. In another study, Mead et al. loaded MSC-derived exosomes with retinal conditioned medium and TNFα, showing significant neuroprotective potential in injured rat models and hRGCs [77]. It is believed that priming with retinal conditioned medium and TNFα helps increase levels of PEDF and VEGF-AA via enhanced abundance in exosomes [77].

Seyedrazizadeh et al. demonstrated that exosomes isolated from human embryonic MSCs (ES-MSCs) improved Brn3a+ RGCs and reduced degenerative thinning of the retinal nerve fiber layer (RNFL) in a C57BL/J6 ONC male mice model [78]. The plausible mechanism behind retinal protection was the reduction of cis-p-tau and blockade of tauopathy, which was prominent in control studies [78]. Seong et al. produced exosome-rich media by inducing hypoxic conditions in amniotic membrane mesenchymal (AMMSCs) and epithelial stem cells (AMESCs), exhibiting protective effects for retinal pigment epithelial cells (RPEC) against hypoxia and H2O2-mediated insults. Intravitreal injections restored intraocular pressure to normal levels within 2 weeks of therapy.

The results of various studies indicate that exosome-based therapies hold promise for the treatment of glaucoma in the near future. However, it's noted that the protective effects are short-term and not effective for longer durations [75, 76]. Recommendations for repeated intravitreal injections to maintain therapeutic levels in circulation may pose challenges due to potential adverse effects, which currently represent a major hurdle in the development of exosome-based therapies.

Diabetic Retinopathy: Diabetic retinopathy, a common comorbidity of diabetes, can lead to blindness [80], with both proliferative and non-proliferative forms capable of causing diabetic macular edema [80]. Exosomes have been investigated for their ability to deliver therapeutic agents or regulatory molecules to control inflammation, vascular dysfunction, and retinal damage associated with diabetic retinopathy (Table 4 provides therapeutic applications) [81].

Exosomes, rich in miRNA contents, play a role in modulating miRNA signaling and protein signaling in retinal cells. A 12-week subconjunctival administration of rabbit adipose MSC-derived exosomes facilitated the regeneration of retinal layers similar to those in a normal retina in a rabbit model [82]. Conversely, intravenous injection of adipose MSC-derived exosomes did not exhibit any recovery, and ganglionic layers remained irregular, similar to the control disease group [82].

Chen et al. reported that exosomes derived from hUCMSCs inhibited hypoxia-induced cell death by increasing the expression of miRNA-21 and inhibiting the p38 MAPK signaling pathway [83]. MSC-conditioned media significantly enhanced exosome marker levels and miRNA levels [83]. Endothelial-to-mesenchymal transition (EndoMT) has been identified as a major contributor to proliferative diabetic retinopathy. Gu et al. found that under high glucose conditions, retinal pigment epithelial cells (ARPE-19) produce miRNA-202-5p-rich exosomes (RPE-Exo), which prevent EndoMT and suppress the growth of human umbilical vein endothelial cells (HUVECs). RPE-Exo, with a high content of miRNA-202-5p, negatively regulates TGFβR2 expression and may be employed for the treatment of diabetic retinopathy [84].

**Table 4:** Therapeutic applications of exosomes in Diabetic retinopathy.

Exosomes source cells	Exosome content	Target	Mechanism of action	Reference
**Adipose MSCs**	miRNAs	MiRNA-222	Retinal regeneration and improve the expression of miRNA-222	Safwat *et al.* [82]
**Human umbilical cord mesenchymal stem cells (hUCMSCs)**	Overexpressed level of exosome markers and miRNAs	p38 MAPK signaling	Inhibit the p38 MAPK signaling and enhance the miRNA-21 level	Chen *et al.* [83]
**Retinal pigment** **epithelial cell line (ARPE-19)**	miRNA-202-5p	TGFβR2	Negatively regulate the TGFβR2 and inhibit the EndoMT	Gu *et al.* [84]
**Bone marrow- derived MSC (BMSC)**	miRNA-486-3p	TLR4 and nuclear factor- kappaB (NF- κB)	Enhanced levels of miRNA-486- 3p while inhibited the TLR4 and nuclear factor- kappaB (NF-κB) expression	Li *et al.* [85]
**RGC-5 and HUVEC**	----	miRNA- 3976	Lower the expression of miRNA-3976 andinhibit the overexpressed NF-κB	Yang *et al.* [86]

In high glucose-treated Muller cells, Li et al. observed downregulation of miRNA-486-3p alongside enhanced expression of TLR4 and nuclear factor-kappaB (NF-κB). Restoration of miRNA-486-3p levels via administration of BMSC-derived exosomes inhibited cellular oxidative stress and apoptosis, further reversing the upregulated expression of TLR4 in Muller cells [85]. Similarly, Yang et al. reported that overexpression of miRNA-3976 in diabetic retinopathy leads to enhanced apoptosis in RGC-5 cells, while treatment with diabetic retinopathy (DR)-derived exosomes reversed this process and improved proliferation in RGC-5 cells. Overexpression of miRNA-3976 results in overexpressed NF-κB signaling, possibly contributing to increased cellular apoptosis [86]. Selvakumar et al. also highlighted the diagnostic application of exosomes in diabetic retinopathy [87], further underlining the significance of exosomes in the development of theranostics or therapeutics in the near future for diabetic retinopathy treatment.

**Table 5:** Therapeutic applications of exosomes in Uveitis.

Exosomes source cells		Exosome content	Target	Mechanism of action	Reference
**Splenic B cells**		IL-35-producing regulatory B-cells	IL-35	Improve the IL-10 and IL-35 levels via increasing the Treg cells and suppress Th17 responses	Kang *et al.* [92]
**Innate cells**	B-1a	Overexpressed IL-27	IL-27 and Treg/ Th17 cells	I27-Bregs secrete IL-27 rich exosomes which improve level of IL-27 and improve Teg/Th17 cell ratio	Kang *et al.* [93]
**Human umbilical MSC**	cord	Overexpressed IL-10	IL-10 and Treg/ Th17 cells	Overexpressed IL-10 improve the Teg/Th17 cell ratio *in vitro* and *in vivo*	Li *et al.* [94]
**Human umbilical MSC**	cord	----	Unknown	----	Bai *et al.* [95]

Uveitis: Uveitis, an autoimmune disease responsible for blindness worldwide [88], involves middle-layer inflammation of the eyes, complicating diagnostic, and clinical therapy efforts (Table 5 provides details) [89, 90]. Exploring exosomal therapy in uveitis could open new avenues for treating this complex autoimmune uveoretinitis (EAU). Research has examined exosomes for their immunomodulatory effects in uveitis, potentially regulating the inflammatory response in the eye. One study on exosomes derived from EAU patients revealed overexpression of miRNA-19b-3p, resulting in an imbalance between Treg/T Helper 17 cell ratio. Inhibition of miRNA-19b-3p in uveitis could be a potential treatment target [91].

Kang et al. generated i35-Breg (IL-35-producing regulatory B-cells) exosomes from splenic B cells and investigated their therapeutic efficacy in autoimmune uveitis. In the EAU model, mice treated with i35-Breg showed potential protection against severe EAU compared to the control group. Mechanistic studies revealed an increase in Treg cells, leading to elevated IL-10 and IL-35 levels [92]. In another study, the same research group reported similar effects with i27-Breg (IL-27-producing B-1a regulatory B cell) in EAU mice [93]. Improving the Treg/Th17 cell ratio serves as a potential target for uveitis treatment. Li et al. administered IL-10 overexpressing MSC-derived exosomes in vitro and in an EAU mice model, finding that these exosomes had a better suppressing effect compared to normal MSC-derived exosomes. The IL-10-based exosomes significantly suppressed T-cell proliferation and Th17 cell differentiation while enhancing Treg cell differentiation [94]. Bai et al. reported that hUCMSC-derived exosomes reversed EAU severity by reducing T cell infiltration and other inflammatory markers, including CCL2 and CCL21 [95].

### Other Ocular Diseases

Exosome-based therapies are extensively explored against a wide range of ocular diseases in this decade [96]. They have been investigated for their potential in treating conditions such as dry eye disease by delivering factors that promote tear production or reduce ocular surface inflammation [97]. Building on the therapeutic efficacy of MSCs in dry eye disease therapy [98], this group-initiated phase 1/2 clinical studies in 2019 to evaluate MSC-derived exosomes' therapeutic potential against chronic graft versus host disease (cGvHD) [99]. Mosseive et al. examined MSC-based exosomes in a retinal ischemia mice model, reporting that exosomes containing miRNAs and paracrine factors positively affected reducing retinal thinning and CNV [100]. Zhou et al. demonstrated that exosomes carrying miRNA-204 ameliorated cGvHD-induced dry eye disease by reprogramming M1 macrophages. Modulation of the IL-6/IL-6R/Stat3 pathway by miRNA-204 played a crucial role in attenuating this disease [101]. The therapeutic potential of MSC-derived exosomes in cGvHD-induced dry eye disease was further validated by Harrell et al. [102].

Mead and Tomarev highlighted the therapeutic potential of Bone marrow-derived MSC (BMSC) based exosomes in glaucoma models [75, 76]. Expanding on these findings, Tassew et al. further discovered that fibroblast-derived exosomes expedite axonal regeneration by activating the autocrine Wnt10b-mTOR pathway in optic neuropathy [103]. Building on this research, Pan et al. investigated umbilical cord MSC (UMSC)-derived exosomes and observed that while these exosomes supported the survival of RGCs and activated glial cells, they did not promote axonal regeneration like BMSC-derived exosomes [104]. The difference in the results between BMSC and UMSC-based exosomes may be attributed to significant differences in their miRNA cargos. Fang et al. reported that UMSC-exosomes are rich in miRNA-21-5p, miRNA-125b-5p, miRNA-23a-3p, miRNA-100-5p, and let-7f-5p [105], while Baglio et al. previously noted that BMSC-exosomes are a rich source of miRNA-143-3p, miRNA-10b-5p, miRNA-486-5p, miRNA-22-3p, and miRNA-21-5p [106].

### Ocular Transplants, Tumors, and Genetic Disorders

Exosomes hold promise in various aspects of ophthalmology, including promoting graft survival and reducing immune rejection in corneal or retinal transplants. In ocular tumors, they could serve as carriers for biomarkers, enabling non-invasive detection, and potentially delivering targeted therapies. Inherited retinal disorders like Leber congenital amaurosis or Stargardt disease are also being targeted for exosome-mediated delivery of gene-editing tools or corrective genetic material. The versatility of exosomes as carriers of therapeutic cargo, their capacity to modulate immune responses, and their ability to transfer molecular information between cells make them an attractive avenue for developing innovative treatments across a range of ocular diseases. Ongoing research and clinical trials continue to explore and refine their use in addressing these diverse ophthalmic conditions.

In conclusion, Exosomes, small vesicles secreted by cells, hold promise in various ophthalmic diseases by transferring molecular cargo between cells. They have been studied extensively for their therapeutic potential in conditions such as age-related macular degeneration (AMD), corneal diseases, glaucoma, diabetic retinopathy, uveitis, and other ocular disorders. Exosomes have shown efficacy in delivering therapeutic molecules to target cells, promoting tissue repair, and modulating immune responses. Ongoing research aims to harness the versatility of exosomes for innovative treatments in ocular diseases, including ocular transplants, tumors, and genetic disorders.

### Research progress of exosomes in ophthalmic diseases

Exosomes hold tremendous potential in various aspects of ocular health, offering promising avenues for both therapeutic interventions and diagnostic applications:

Regenerative Potential and Corneal Healing: Exosomes derived from different cell types, especially mesenchymal stem cells (MSCs), demonstrate regenerative effects, fostering corneal wound healing, reducing scarring, and enhancing transparency. These findings suggest potential applications in treating corneal injuries and disorders. [107].

### Neuroprotection in Optic Nerve Diseases

Studies indicate that exosomes possess neuroprotective properties in conditions affecting the optic nerve, such as optic neuritis or ischemic optic neuropathy. They may help mitigate inflammation, support neuronal survival, and potentially alleviate optic nerve damage [104].

### Inflammatory Eye Diseases

Exosomes carrying immunomodulatory cargo are under investigation for inflammatory eye conditions like uveitis and ocular surface diseases. They offer the potential to regulate immune responses, modulate inflammatory pathways, and facilitate tissue repair. Ongoing research focuses on utilizing exosomes as novel treatment modalities for various eye disorders. Strategies include modifying exosomes for targeted drug delivery, engineering them with specific cargo, or exploring combination therapies to enhance treatment efficacy [108].

### Diagnostic Biomarkers

Profiling exosome cargo from ocular fluids for disease-specific biomarkers holds promise for conditions such as retinal degeneration, glaucoma, and age-related eye diseases. Identifying unique molecular signatures in exosomes may enable the development of non-invasive diagnostic tools [109].

### Retinal Diseases and Neuroprotection

Investigations into exosomes' potential in retinal diseases like age-related macular degeneration (AMD) and diabetic retinopathy continue. Exosomes derived from various sources aim to preserve retinal function and slow degenerative processes. [110].

### Retinal Detachment

Research explores the involvement of exosomes in retinal detachment, focusing on their roles in inflammation, cellular damage, and potential contributions to promoting retinal regeneration [96].

### Customized Cargo Loading

Efforts are underway to refine methods to modify exosome cargo for therapeutic purposes, including loading them with specific drugs, genetic material, or growth factors to enhance their efficacy in treating ocular diseases [111].

### Exosomes in Optic Nerve Regeneration

Exosome-based approaches for optic nerve regeneration, particularly after injury or in conditions like optic neuropathies, aim to support axonal regrowth and neuronal survival, offering new treatments for these conditions [112].

### Combination Therapies and Drug Delivery Systems

Explorations into combining exosome-based therapies with other treatment modalities or using exosomes as drug delivery systems seek to enhance therapeutic outcomes and broaden the scope of treatments for various ocular diseases. [113].

### Exosome Mimetics and Nanovesicles Development

The development and refinement of synthetic exosome mimetics or nanovesicles aim to replicate the functions of natural exosomes with improved control over cargo loading and targeting capabilities, offering potential alternatives for therapeutic interventions [114].

In conclusion, exosomes, particularly those derived from mesenchymal stem cells (MSCs), hold regenerative potential for corneal healing, reducing scarring, and enhancing transparency, suggesting promising applications in treating corneal injuries.

## List of exosomes currently in clinical trials

Exosomes, as carriers, offer distinct advantages over other drug delivery systems. Their nanoscale size, coupled with low immunogenicity, minimal biodegradation, and extended circulation time, enhances cellular uptake—a key distinguishing factor from alternative carriers [54, 115]. Studies have consistently shown that exosomes loaded with drugs outperform free drugs or control exosomes, demonstrating superior efficacy and bioavailability [55, 56]. Moreover, exosomes exhibit the remarkable ability to traverse biological barriers, positioning them as pivotal players in the advancement of drug delivery and biomarker identification. Rich in nucleic acids, proteolipids, and immune factors, exosomes facilitate repair, regeneration, and information transfer within the body [116, 117]. Their tissue-specific cargo content holds promise for disease-specific biomarker identification. Engineered structures and site-specific delivery mechanisms further enhance exosomes' efficacy over conventional carriers, garnering significant attention from researchers, particularly in ocular disease therapeutics [99]. The burgeoning field of exosome-based nano-drug delivery systems heralds a paradigm shift in pharmaceutical formulation for targeted therapy, with numerous therapeutic exosomes undergoing clinical trials for various ocular diseases, as outlined in Table 5. Larger-scale exosome production is accomplished using formats such as dozens of large flasks (e.g., T-225), multiple stacked array multilayer culture flasks, large fixed-bed bioreactors, stirred-tank bioreactors with microcarriers, or continuous production in perfusion reactors. First, it should be noted that all the issues of modern technical transfer exist when developing a large-scale, clinically relevant exosome manufacturing format. This includes all the steps involved in transitioning from an investigational product to a commercial product, moving from laboratory-scale production to commercial scale, and implementing a comprehensive quality control testing program. Steps include process optimization, confirmation, validation, and characterization, as well as process performance qualification activities. Additionally, many newer and higher industry goals will apply during this transfer. These goals include enhanced process control, establishing as many closed operations as possible, moving to more automated and digital processes, employing single-use systems, and implementing extensive in situ monitoring devices and sample analytics. Due to the poor efficiency of in vitro exosome production, scaling up standard batch-mode manufacturing can require the use of hundreds of flasks or a significant investment in more expensive and complex multilayer flask systems. Challenges associated with these approaches include the cost of culture expansion before the actual production phase, which may involve stem cell-conditioned medium, or the additional cost and timing required to begin the production phase in serum-adjusted or specialty serum-modified medium. Robust production of consistently homogeneous populations of exosomes requires verification of reproducible culture conditions, which can take time to establish during the scale-up or scale-out of such systems [119].

In conclusion, exosomes stand out as drug carriers due to their nanoscale size, low immunogenicity, extended circulation time, and high cellular uptake, offering advantages over other carriers. Studies have demonstrated that exosomes loaded with drugs exhibit enhanced efficacy and bioavailability compared to free drugs. However, scaling up exosome production poses challenges, often requiring complex and costly equipment such as large flasks or bioreactors.

## Conclusion

In recent years, research into utilizing extracellular vesicles, particularly exosomes, for targeting ophthalmic diseases has unveiled promising potential. Exosomes, small vesicles released by cells containing biomolecules such as proteins, nucleic acids, and lipids, stand as key candidates for therapeutic use across various ophthalmic conditions. The focus lies on assessing the long-term safety and feasibility of exosome-based therapies for clinical applications in ophthalmology. Early-phase clinical trials aim to evaluate safety and initial efficacy in human subjects. The research endeavors to deepen understanding of exosome biology, encompassing cargo modification techniques, effects on angiogenesis, and the development of synthetic exosome mimetics. These efforts aim to enhance therapeutic potential and specificity of exosome-based interventions. Customizing exosome treatments according to individual disease characteristics and improving stability and bioavailability in ocular tissues are actively explored. Further studies concentrate on comprehensive assessments of long-term safety, immune responses, and sustained efficacy of exosome-based therapies in ocular diseases, crucial for broader clinical adoption. Standardizing isolation and purification methods for exosomes is another focus, aiming to enhance reproducibility, scalability, and consistency of exosome-based therapies for successful translation into clinical practice. In essence, current insights suggest that exosomes offer additional advantages in delivering therapeutic payloads, potentially reducing side effects and improving treatment efficacy. They can transport various bioactive molecules, including microRNAs, growth factors, and antioxidants, thereby modulating cellular processes and promoting tissue repair in the eye.

In sum, this review showed the potential of exosomes for eye diseases, and they have significant potential for this purpose. This review highlights the immense research opportunities available to understand the physiological roles and clinical potential of exosomes in eye diseases. Although progress has been made, further studies are needed to advance this field. In addition to original research, we recommend conducting systematic reviews on the effects of exosomes in eye diseases.
